# Detection of genes involved in bud phenology in sessile oak (*Quercus petraea* Matt. Liebl) combining digital expression analysis and Q-PCR

**DOI:** 10.1186/1753-6561-5-S7-P20

**Published:** 2011-09-13

**Authors:** Saneyoshi Ueno, Christophe Klopp, Céline Noirot, Valérie Léger, Elodie Prince, Antoine Kremer, Christophe Plomion, Grégoire Le Provost

**Affiliations:** 1 Forestry and Forest Products Research Institute, Brazil; 2 Plateforme bioinformatique Genotoul, UR875 Biométrie et Intelligence Artificielle, INRA, Brazil; 3 INRA, UMR 1202, BIOGECO, Brazil

## Background

In temperate zones, the seasonal cycling of perennials comprises two main steps: a growing period when environmental conditions are favourable and a non-growing period in winter. Winter dormancy consists of two phases: endodormancy and ecodormancy [1]. The former is the deepest state of the dormancy controlled by the meristem itself, while the latter is controlled by environmental factors, such as accumulated temperatures.This phenological cycle has been shown to be strongly affected by climatic change: higher temperature having a positive effect on ecodormancy phase by accelerating meristem cell growth and early bud break is particularly crucial to consider not only in respect to increasing length of the growing season, but also regarding the risk of frost damage. For these reasons, understanding the molecular mechanism controlling the shift from endo- to ecodormancy is of main importance to optimize the management of forest tree plantations to their most likely environmental conditions.

The objective of this study was to characterize the transcriptome of endo- and eco-dormant sessile oaks (a major broadleaf species in Europe) by RNA-seq and to identify differentially expressed genes between these two dormancy stages. Four cDNA libraries were generated using total RNA extracted from apical buds harvested during endo- or ecodormancy of two contrasted populations. Transcriptome characterization was performed on a Roche 454 FLX platform. Digital expression analysis leads us to identify a set of 48 genes differentially regulated between these two developmental phases (there was no differences between populations). Their function was identified by sequence homology and this result contributes to the characterization of the molecular network underlying vegetative bud dormancy, an important life history traits of these long lived organisms.

## Methods

Acorns of sessile oak (*Quertus petreae* (Matt) Lieb.) were collected in 2006 on two populations from Northern France: Longchamp (LC) and Saint Jean (SJ). These populations are contrasted for their bud burst date (early vs. late flushing). We obtained 560 and 468 seedlings for the SJ and the LC populations, respectively. A forcing test experiment was performed in a greenhouse from July 30th 2007 to February 25th 2008 located at the INRA forestry research station (southwestern France). For each population, approximately 25 seedlings were selected each week. Ten apical buds from 10 seedlings were used for RNA extraction. The 15 remaining seedlings were transferred in a greenhouse with the following parameters: a 16-hours photoperiod and a day/night temperature of 25/18 °C. For each batch of seedlings, bud burst date was evaluated three times a week allowing us to determine the number of days necessary to induce bud burst. We, thus, identified periods corresponding to eco- and endodormancy.

Total RNA from apical buds harvested on September 17th, 24th and October 1st for endo-dormancy as well as on January 14th, 28th and February 11th for eco-dormancy were extracted independently, and mixed equimolarly to obtain a composite cDNA library for each population and developmental stage. The libraries were sequenced on a Roche 454 FLX to produce 495,915 reads in total. After cleaning and removal of duplicated reads, reads were mapped onto oak unigenes [2]. A digital gene expression analysis was performed using both *r* statistics [3]and R BioConductor packages (edgeR, DEGseq and goseq). Homology searches were carried out by BlastX against SWISS-PROT and TAIR9 databases with e-value cutoff of 1e-5. Gene ontology (GO) annotations were based on the top blast hit against SWISS-PROT database.To validate bioinformatic analysis, Q-PCR was performed for 13 selected genes. One microgram of total RNA was reverse transcribed using Improm-II^TM^ reverse transcription system (Promega) according to the manufacturer’s instructions. Q-PCR reaction and quantification were performed on a Chromo4 Multicolor Real-Time PCR Detection System (Bio-Rad)

## Results and conclusions

We identified 48 common contigs that showed differential expression by three bioinformatic programs (R statistics, edgeR and DEGseq) and selected 13 genes for Q-PCR analysis with the main goal to validate the result obtained in silico. One gene (heat shock protein) showed multi-banding pattern and discarded from the analysis. Among the 12 remaining genes, only one gene (cold shock protein) showed contradictory expression pattern between in silico and Q-PCR analysis. The high success rate of Q-PCR analysis (11/13 = 84.6 %) indicates that the verified genes are likely to represent endo- and ecodormancy stages. Two genes (Globulin and XET) were confirmed as up-regulated in endodormancy, while nine genes (GASA, GST, DRM1, XERO2, PIP, AWPM, GID, LEA and ELP) were found to be up-regulated in ecodormancy .Analysis in terms of GO showed genes responsible to abiotic and endogenous stimulus as well as stress related were enriched in the set of 48 genes. Similar trend was observed in expression analysis for raspberry bud dormancy, where high percentage of stress-response/defense/detoxification-related genes were identified [4]. In grape also, most of genes expressed in buds at the end of dormancy were attributed to the oxidative processes and stress responses [5]. Although the number of genes indentified is small and further verification through time course change in their expression will be needed, these candidates will serve as stepping stone to dissect the molecular mechanisms involved in bud dormancy in forest trees.
